# Bibliometric Analysis of Early COVID-19 Research: The Top 50 Cited Papers

**DOI:** 10.1177/1178633720962935

**Published:** 2020-10-13

**Authors:** Hassan ElHawary, Ali Salimi, Nermin Diab, Lee Smith

**Affiliations:** 1 Division of Plastic and Reconstructive Surgery, Faculty of Medicine, McGill University, Montreal, QC, Canada; 2 Department of Ophthalmology, Faculty of Medicine, McGill University, Montreal, QC, Canada; 3 Division of Respirology, Faculty of Medicine, University of Toronto, Toronto, ON, Canada; 4 The Cambridge Centre for Sport and Exercise Sciences, Anglia Ruskin University, Cambridge, UK

**Keywords:** COVID-19, Bibliometric analysis, Evidence-based research

## Abstract

**Introduction:**

The COVID-19 pandemic is rapidly evolving with the number of cases exponentially rising. The research scientific community has reacted promptly as evidenced by an outstanding number of COVID-19 related publications. As the number of scientific publications rapidly rises, there is a need to dissect the factors that lead to highly impactful publications. To that end, the present paper summarizes the characteristics of the top 50 cited COVID-19-related publications that emerged early during the pandemic.

**Methods:**

A systematic search of the Web of Science, Scopus, and Google Scholar was performed, using keywords related to COVID-19 and SARS-CoV-19. Two independent authors reviewed all the search results, screening for the top 50 cited COVID-19-related articles. Inclusion criteria comprised any publication on COVID-19 or the SARS-CoV-2 virus. Data extracted included the type of study, journal, number of citations, number of authors, country of publication, and study content.

**Results:**

As of May 29th, the top 50 cited articles were cited 63849 times during the last 4 months. On average, 14 authors contributed to each publication. Over half of the identified articles were published in only 3 journals. Furthermore, 42% and 26% of the identified articles were retrospective case series and correspondence/viewpoints, respectively, while only 1 article was a randomized controlled trial. In terms of content, almost half (48%) of the identified publications reported clinical/radiological findings while only 7 out of the 50 articles investigated potential treatments.

**Conclusion:**

By highlighting the characteristics of the top 50 cited COVID-19-related articles, the authors hope to disseminate information that could assist researchers to identify the important topics, study characteristics, and gaps in the literature.

## Introduction

December 2019 witnessed the first presentation of pneumonia-like illness that was soon after attributed to the novel SARS-CoV-2 virus.^1^ The Corona Virus Disease (COVID-19) aggressively spread throughout China in the following several weeks and became a world-wide pandemic affecting almost every country in the world within the subsequent 4 months.^2^ More worryingly, recent evidence shows that almost 20% of COVID-19 patients will require hospitalization, causing an enormous burden on health care systems across the world.^3^ As of May 3rd 2020, over 3.5 million cases tested positive with the Corona Virus Disease (COVID-19) of which over 250 000 had a fatal outcome.^2^

While the numbers continue to rise, the scientific research community has promptly responded, with over 500 COVID-19-related clinical trials currently underway.^4^ Research and scientific inquisitions are of paramount importance in the face of pandemics such as COVID-19. The absence of clinically proven curative treatments or vaccines coupled with a paucity in our understanding of the disease and long term clinical sequalae fuels the need for further research to help us better combat the current pandemic.

Bibliometric analyses assess the current status and trends in a specific research domain. This allows us to identify areas of importance and potential gaps in the literature which helps provide ideas and directions for future research. Such studies have been conducted in many different surgical and non-surgical domains; however, none have been performed on COVID-19 related publications.^5-8^ With respect to the COVID-19 pandemic, previous bibliometric analyses have been conducted to investigate research activity in different counties.^9-12^ However, none were conducted with the goal of highlighting the most highly cited COVID-19 early research publications. With the exponential growth of COVID-19-related literature, a bibliometric analysis of the top 50 cited COVID-19 related research is warranted.

To that end, the goal of this study was to present a bibliometric analysis to identify and dissect the characteristics of the top 50 cited COVID-19-related articles published early on following the outbreak. This will provide a deeper understanding of the current COVID-19 research milieu while highlighting potential patterns that could assist future researchers in their scientific pursuits.

## Materials and Methods

### Search strategy

A systematic search of the Web of Science, Scopus, and Google Scholar was performed, using keywords related to COVID-19 and SARS-CoV-19. The search strategy included the following key terms (2019-nCoV) OR (SARS-CoV-2) OR (corona virus) OR (COVID-19) and their associated variations. Search results were limited to 2019-2020. The search was conducted on, and included all articles up until May 29th 2020. The initial search yielded a total of 35 042 results.

### Data collection and synthesis

The results from each database were sorted according to the citation count. The top 200 most cited publications from each search engine were exported to Microsoft Excel. Duplicate entries were manually identified and extracted by 2 independent authors. The top 50 most cited non-duplicate COVID-19 related publications were then identified. Inclusion criteria comprised any publication on COVID-19 or the SARS-CoV-2 virus. Publications in all languages were included. The only exclusion criteria were publications on other coronavirus diseases such as SARS. Data extracted included the type of study, journal, number of citations, number of authors, country of publication, and study content. All descriptive statistics were performed using Microsoft Excel functions.

## Results

As of May 29th 2020, the top 50 cited COVID-19 related articles were cited a total of 63 849 times, with an average of 1277 (SD: 1084) per article.^2,13-61^ Over a third of the articles (n = 19) had more than 1000 citations and 18% (n = 9) had more than 2000 citations. The highest cited article was published in The Lancet and was cited 5897 times in a span of less than 5 months.^26^ As one of the earliest cohort studies on COVID-19’s clinical presentations, this study reported the clinical findings as well as patient characteristics of 41 COVID-19 patients in Wuhan.

The top 50 cited publications were published in 22 journals. Over half (n = 26) were published in only 3 journals: The Lancet, the New England Journal of Medicine, and the Journal of American Medical Association (JAMA). More interestingly, these 26 articles were cited a total of 44117 (69.1% of the total number of citations). The number of authors on each article varied from 2 to 65 authors with a mean of 14 (SD:12.2) authors. Furthermore, the most common types of articles were retrospective case series (42%) and correspondences/viewpoints (26%) while only one publication was a randomized controlled trial (RCT).

**Figure 1.**
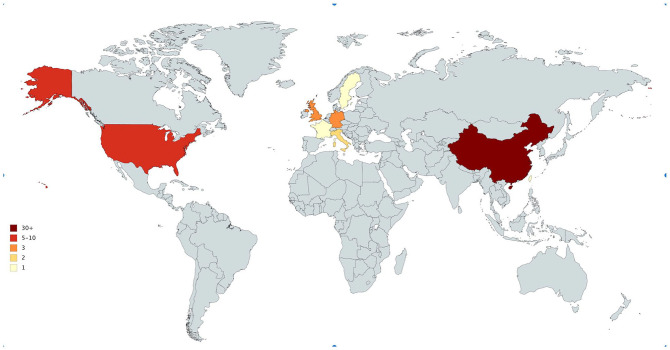
Top 50 cited COVID-19 related articles’ country of origin.

The top 50 cited articles were published out of 8 counties. China and the United States contributed to the majority of the publication (n = 31 and n = 8, respectively). Germany and the United Kingdom contributed to 3 publications each. Italy contributed to 2 publications while the remaining countries all contributed to 1 publication each (Figure 1). In terms of content, 48% of the articles (n = 24) reported clinical/radiological findings, 18% (n = 9) discussed basic science/genomic characterizations of the virus; and only 14% (n = 7) discussed treatment options (Table 1).

**Table 1.** Characteristics of the top 50 cited COVID-19-related publications.

**Journal**	**Number of** p**ublications**	**Total** c**itations**	**Number of** e**ach** p**ublication type**					**Publication** c**ontent**					
			Retrospective/case series	Correspondence/letter to the editor***	Trials*	Review	Forecasting/modeling	Clinical findings	Public health/epidemiology	Transmission	Basic science/genomic characterization	Treatment	Other
***The Lancet***	12	20 508	6	3	0	1	2	5	3	1	1	1	1
***New England Journal of Medicine***	8	15 177	4	3	1	0	0	5	0	2	0	1	0
Lancet Associated Journals*	5	4167	3	2	0	0	0	4	1	0	0	0	0
***JAMA/JAMA Internal Medicine***	6	8432	3	3	0	0	0	5	0	0	1	0	0
***Nature/Nature Reviews***	3	3986	2	0	0	1	0	0	0	0	3	0	0
***International Journal of Antimicrobial Agents***	2	1590	0	0	1	1	0	1	0	0	0	1	0
***Science***	2	1470	0	0	0	0	2	0	1	0	1	0	0
Others**	12	8519	3	2	0	2	5	4	0	1	3	4	0
**Total**	50	63 849	21	13	2	5	9	24	5	4	9	7	1

* Lancet associated journals include *Lancet Respiratory Medicine, Lancet Infectious Disease* and *Lancet Global Health*.

** Other journals include *American Journal of Obstetrics and Gynecology, Annals of Internal Medicine, Bioscience Trends, Eurosurveillance, Intensive Care Medicine, International Journal of Infectious Disease, Journal of Autoimmunity, Journal of Korean Medical Science, Journal of Travel Medicine, Journal of Virology, Science, Radiology*.

*** Correspondence/letter to the editor additionally include viewpoints, editorials and comments.

## Discussion

The current paper presents a bibliometric analysis of the top 50 cited articles related to COVID-19. Our analysis assesses the current status and trends of early COVID-19 research and highlights several interesting themes.

The first of which is that the scientific community’s response to the current COVID-19 pandemic was prompt and rigorous as evidenced by the outstanding number of citations that the identified articles received over the last 4 months. The open access policy that many journals have implemented with regards to COVID-19 publications has potentially contributed to quick dissemination of information and the exponential growth of publications in a short period of time.

The top 50 articles were published in 22 journals. Among these journals are the highest 7 out of 10 (including the top 2) impact factor journals across all domains. Approximately a quarter (n = 12) of the top 50 studies were published in 1 journal (The Lancet) and over half (n = 26) were published in 3 journals (The Lancet, New England Journal of Medicine, and JAMA). The distribution of the publications among these journals is consistent with Bradford’s law which stipulates that if you sort the journals by the number of articles they publish into 4 groups, each with about a quarter of the number of publications, then the number of journals in each group will be proportional to 1:2:2^2^:2^3^.^62^

The majority of the highly cited research assessed COVID-19’s clinical presentation and disease description while only 7 papers discussed potential treatment. This could be explained by the current limitation in our understanding of the SARS-CoV-2 virus and the need to better understand its associated disease before being able to investigate potential treatments. However, as the medical domain gets more accustomed to COVID-19, future studies should examine the efficacy of various treatments and vaccines.

Our analysis showed that on average each research article was conducted by 14 authors, which demonstrates the value of collaboration especially in high acuity situations such as pandemics. In addition, the results of this report demonstrate that the majority of highly cited COVID-19-related research were retrospective case series or commentaries/correspondences. While randomized controlled trials remain the gold standard of scientific research, due to the rapidly evolving nature of the situation and the short time period since the inception of the pandemic, lower grade evidence from retrospective research is of great value and can provide important information to help us better characterize the disease and its clinical presentation. The authors believe that higher-level evidence from controlled trials is still warranted to further our understanding of this disease.

This study has some limitations. First, the authors did not search all scientific databases. However, to address this limitation and ensure maximal inclusivity, the authors searched 3 of the most commonly used databases. Moreover, a limitation to any bibliometric analysis is the fact that citation frequency is affected by multiple factors such as journal and institution reputation and therefore is not a perfect reflection for academic influence. Furthermore, due to the short period of time since the inception of the pandemic and the quickly evolving nature of COVID-19 research, the number of citations will change over time. While this limitation is present with any bibliometric analysis, the main goal of this study was to highlight the characteristics of the highly cited research articles early during the COVID-19 pandemic and the dynamic nature of citation count should not diminish the value of the information presented here.

## Conclusion

The current study presents a concise bibliometric analysis of the current COVID-19 research milieu. Our study shows that the scientific community has been very rigorous and active in publishing COVID-19-related articles as evidenced by the enormous number of citations the top 50 articles received, within less than half a year following the emergence of this disease. The majority of these papers are published among the most reputable journals world-wide. With the majority of the top 50 articles assessing COVID-19’s clinical/radiological findings, there remains a paucity in articles investigating treatments/vaccines.
